# Process Parameters Optimization and Mechanical Properties of Additively Manufactured Ankle–Foot Orthoses Based on Polypropylene

**DOI:** 10.3390/polym17141921

**Published:** 2025-07-11

**Authors:** Sahar Swesi, Mohamed Yousfi, Nicolas Tardif, Abder Banoune

**Affiliations:** 1 Université de Lyon, CNRS, UMR 5223, Ingénierie des Matériaux Polymères, Université Claude Bernard Lyon 1, INSA Lyon, Université Jean Monnet, 69621 Villeurbanne Cedex, France; sahar.swesi@insa-lyon.fr; 2 Univ Lyon, INSA-Lyon, CNRS, LaMCoS, UMR5259, 69621 Villeurbanne, France; nicolas.tardif@insa-lyon.fr; 3 Handicap International, Humanité et Inclusion, 69008 Lyon, France; a.banoune@hi.org

**Keywords:** polypropylene, fused filament fabrication, optimization, Taguchi design of experiments, process simulation, warpage, residual stresses

## Abstract

Nowadays, Fused Filament Fabrication (FFF) 3D printing offers promising opportunities for the customized manufacturing of ankle–foot orthoses (AFOs) targeted towards rehabilitation purposes. Polypropylene (PP) represents an ideal candidate in orthotic applications due to its light weight and superior mechanical properties, offering an excellent balance between flexibility, chemical resistance, biocompatibility, and long-term durability. However, Additive Manufacturing (AM) of AFOs based on PP remains a major challenge due to its limited bed adhesion and high shrinkage, especially for making large parts such as AFOs. The primary innovation of the present study lies in the optimization of FFF 3D printing parameters for the fabrication of functional, patient-specific orthoses using PP, a material still underutilized in the AM of medical devices. Firstly, a thorough thermomechanical characterization was conducted, allowing the implementation of a (thermo-)elastic material model for the used PP filament. Thereafter, a Taguchi design of experiments (DOE) was established to study the influence of several printing parameters (extrusion temperature, printing speed, layer thickness, infill density, infill pattern, and part orientation) on the mechanical properties of 3D-printed specimens. Three-point bending tests were conducted to evaluate the strength and stiffness of the samples, while additional tensile tests were performed on the 3D-printed orthoses using a home-made innovative device to validate the optimal configurations. The results showed that the maximum flexural modulus of 3D-printed specimens was achieved when the printing speed was around 50 mm/s. The most significant parameter for mechanical performance and reduction in printing time was shown to be infill density, contributing 73.2% to maximum stress and 75.2% to Interlaminar Shear Strength (ILSS). Finally, the applicability of the finite element method (FEM) to simulate the FFF process-induced deflections, part distortion (warpage), and residual stresses in 3D-printed orthoses was investigated using a numerical simulation tool (Digimat-AM^®^). The combination of Taguchi DOE with Digimat-AM for polypropylene AFOs highlighted that the 90° orientation appeared to be the most suitable configuration, as it minimizes deformation and von Mises stress, ensuring improved quality and robustness of the printed orthoses. The findings from this study contribute by providing a reliable method for printing PP parts with improved mechanical performance, thereby opening new opportunities for its use in medical-grade additive manufacturing.

## 1. Introduction

Worldwide, over 40 million people require assistive devices such as prostheses and orthoses, yet access to such equipment remains extremely limited in low- and middle-income countries, where only 5 to 15% of those in need can benefit from technical support, according to the World Health Organization (WHO) [1]. In response to this critical issue, the NGO Humanity & Inclusion (also known as Handicap International, HI) has been promoting the use of additive manufacturing (AM) since 2017 to improve the availability and personalization of orthopedic devices in low-resource settings [2]. Among various AM technologies, Fused Filament Fabrication (FFF) stands out for its cost-effectiveness, ease of implementation, and ability to fabricate patient-specific orthoses directly from digital models [3,4].

Polypropylene (PP), a semi-crystalline thermoplastic polymer, has been adopted by several manufacturers for its favorable balance between mechanical performance, flexibility, and chemical resistance, making it particularly suitable for ankle–foot orthoses (AFOs) [5,6,7]. However, its processing by FFF remains challenging due to its low adhesion to the print bed, high shrinkage, and tendency to warp during cooling [8].

Compared to other thermoplastics, polypropylene was chosen specifically for its superior mechanical and chemical properties that are highly suitable for orthotic applications requiring controlled flexibility and fatigue resistance, which are crucial for orthoses intended for prolonged use [9,10]. PP was preferred among other polymers such as poly(Lactic Acid) (PLA), Poly Ethylene Terephthalate Glycol (PETG), or poly (Acrylonitrile-Butadiene Styrene) (ABS) for its light weight, contributing to patient comfort, and for its biocompatibility and resistance to moisture and chemicals, enabling hygienic use and easy cleaning [7]. PP was also chosen for its long-term durability [11,12]. While PLA, PETG, and ABS are more commonly used due to their easier printability, they have significant limitations for medical and orthotic purposes. PLA is brittle, sensitive to heat and moisture, and unsuitable for wearable applications requiring mechanical flexibility. PETG offers better strength than PLA but lacks the flexibility and resilience needed for patient comfort. ABS, while stronger, releases potentially harmful fumes during printing and is less skin-friendly for long-term wear [13].

Several articles have been reported that use FFF 3D printing, particularly in the production of ankle–foot orthoses (AFOs) [14,15,16]. An important aspect to consider when choosing the material for orthoses is stiffness. According to Totah et al. [17,18], the stiffness of an AFO should be between 2 Nm/deg and 10 Nm/deg to meet the comfort and support criteria required for wearing an orthosis. According to Delbruel et al. [18], polypropylene material falls within this range, allowing a compromise to be satisfied between flexibility and mechanical resistance. In this context, optimizing the printing process of PP-based AFOs is essential to ensure consistent part quality and mechanical reliability. Moreover, a deeper understanding of the relationship between printing parameters and material behavior is required to adapt this technology to humanitarian field conditions, where production speed, simplicity, and robustness are key constraints.

Optimization of FFF process parameters based on the Taguchi method has been widely deployed in the literature to obtain parts with optimal mechanical properties and develop high-performance designs of prototypes. Sharma et al. [19] used the Taguchi method as a powerful tool to screen significant process parameters for FFF 3D printing of PLA parts while preparing a significantly smaller number of experiments.

It is worth noting that the primary innovation of our study lies in the optimization of 3D printing parameters for polypropylene (PP) in the fabrication of customized orthoses, addressing the well-known challenges associated with this material cited above (such as shrinkage, poor interlayer adhesion, and limited bed adhesion).

Our work proposes to produce functional, patient-specific devices using PP, a material still underutilized in the additive manufacturing of medical devices. A combined experimental and numerical approach is proposed: first, a thermomechanical characterization of the PP filament is conducted to calibrate a material model; second, a Taguchi design of experiments explores the impact of key printing parameters (e.g., extrusion temperature, printing speed, infill pattern) on the mechanical properties of printed parts. Finally, the process-induced defects, such as warpage and residual stresses, are analyzed through finite element simulations using Digimat-AM^®^. The overall aim is to define a robust printing configuration that enhances mechanical performance while remaining suitable for deployment in humanitarian contexts.

## 2. Materials and Methods

### 2.1. Materials and Equipment

In this study, TREED PLENE 5 polypropylene (PP) filament (Treed, Seregno, Italy), manufactured from a commercial PP with the reference Hifax 3080 (LyonDellBasell, Ferrara, Italy), was used for FFF 3D printing. The 3D printer used for printing the orthoses and bending bars was the WASP 4070 ZX (WASP, Massa Lombarda, Italy), a Delta-type 3D printer. It has a print volume of 400 × 400 × 700 mm and operates with a delta kinematic system, where the three axes move independently using stepper motors.

The parts were either designed using computer-aided design (CAD) software (Catia V6, Dassault Systemes, Vélizy-Villacoublay, France) or obtained through reverse engineering with OrtenShape software (Proteor, Dijon, France) [20]. They were then exported in STL format and prepared for printing using the Ultimaker Cura slicing software (Ultimaker, Geldermalsen, The Netherlands). After setting the printing conditions, the generated G-code file was transferred to the 3D printer for fabrication.

### 2.2. Characterization Methods

The first step of this study consisted of characterizing the PP to evaluate its thermal and mechanical properties. These data will serve as the basis for creating the material in the Digimat 2022.4 database software (MSC Software, Mont-Saint-Guibert, Belgium) and simulating the printing of the orthoses.

#### 2.2.1. Differential Scanning Calorimetry

DSC analysis was performed to verify whether the TREED PLENE 5 filament exhibits a structure and thermal behavior similar to the Hifax 3080 granules. Two samples were prepared: one from the TREED PLENE 5 filament and the other from the Hifax 3080 granules, with respective masses of 6.07 mg and 8.50 mg, placed in hermetically sealed crucibles. An empty reference was used for the measurement. The analysis was carried out using a differential scanning calorimeter (DSC Q20 and Q23, TA Instruments, New Castle, DE, USA). The thermal protocol followed was as follows:

- Heating the sample from −60 °C to 210 °C, followed by a 1 min isotherm at 210 °C to erase thermal history.
- Cooling to −60 °C.
- Second heating cycle up to 210 °C.
- Final cooling to 25 °C before ending the analysis.

The heating rate was set at 10 °C/min under a nitrogen atmosphere to prevent any thermal degradation. The analysis was conducted on the second cycle to eliminate the effects of initial thermal stresses.

From the obtained curves, the melting and crystallization characteristic temperatures of both samples were compared. The crystallinity rate was calculated using the following equation:

(1) $$X_{c}=\frac{{\Delta H}_{f}}{{\Delta H}_{f}^{0}\times x_{PP}}\times 100\;$$

where ${\Delta H}_{f}$ is the measured enthalpy of fusion of the sample, ${\Delta H}_{f}^{0}$ is the theoretical enthalpy of fusion of 100% crystalline PP (taken as 207 J/g), and $x_{PP}$ is the mass fraction of PP.

#### 2.2.2. Measurement of Specific Heat Capacity as a Function of Temperature

To determine the specific heat capacity (*Cp*) of PP as a function of temperature, a heat flow DSC analysis can be performed. This method allows the specific heat of the material to be measured over a given temperature range. The heat capacity is calculated based on heat flow, heating rate, and a calibration constant (*E*) of the device using the following equation:

(2) $$C_{P}=\frac{\left(\frac{dH}{dt}\right)}{m\times (\frac{dT}{dt})}\times E\;$$

The final equation used to calculate the specific heat capacity is as follows:

(3) $$Cp=\frac{E\times H\times 60}{H_{r}\times m}\;$$

where *Cp* is the specific heat (J/g°C), *E* is the calibration constant (dimensionless), H is the heat flow (mW), $H_{r}$ is the cooling rate (°C/min), and m is the sample mass (mg).

The thermal protocol to be followed is as follows:

- Stabilizing the sample temperature at 20 °C.
- Heating the sample from 20 °C to 210 °C at 20 °C/min, followed by a 1 min isotherm at 210 °C.
- Cooling back to 20 °C at 20 °C/min.

Initially, measurements are performed with empty crucibles to generate a baseline. Once the baseline is obtained and corrections applied, the same protocol is used to measure the heat capacity of a sample mass *m*. The results are analyzed by comparing the heat flow of the baseline and the sample, accounting for the calibration constant *E*. The baseline is then subtracted from the sample measurements. Finally, the heat capacity can be calculated using Equation (2).

#### 2.2.3. Thermogravimetric Analysis

A thermogravimetric analysis (TGA) was carried out to measure the variation in mass of a sample as a function of temperature, thus enabling the study of thermal stability and material degradation. This method is essential for evaluating the thermal stability of our samples and determining their behavior during heating up to degradation. The analysis also allows for detecting the presence of mineral fillers in the material, as these do not degrade under heat.

The analysis was performed using a TGA Q500 thermogravimeter, TA Instruments, New Castle, DE, USA, on Hifax 3080 granules. The samples were weighed and placed in platinum and alumina pans. Before any measurement, the pans were carefully cleaned using a torch to remove residues.

The thermal program was as follows: temperature ramp from 30 °C to 800 °C at a heating rate of 10 °C/min. The test was conducted under non-dehydrated nitrogen, with a gas flow of 10 mL/min for the balance and 90 mL/min for the sample.

Mass loss (*ML*) during the test can be calculated using the following formula:

(4) $$M_{L}=\frac{m_{start}-m_{end}}{m_{start}}\times 100$$

where $m_{start}$ is the mass at the beginning of degradation and $m_{end}$ the mass at the end of degradation.

Furthermore, the thermal stability index (*TSI*) can be calculated using the following formula:

(5) $$TSI=\frac{T_{start}}{T_{end}}$$

The closer the *TSI* value is to 1, the more stable the material is considered to be, with slow and gradual degradation.

#### 2.2.4. Thermomechanical Analysis

A thermomechanical analysis (TMA) was performed to measure the expansion or contraction of the material as a function of a defined thermal profile. This analysis enables the determination of the coefficient of thermal expansion (CTE) of PP.

The test was conducted on an injected PP Hifax 3080 bar with a thickness of 2.06 mm. The machine used was the Universal V4.5A from TA Instruments, New Castle, DE, USA. The analysis was carried out in macro-expansion mode under a nitrogen atmosphere (50 mL/min) to limit oxidation of the material during heating.

The experimental protocol involved subjecting the sample to a progressive temperature increase while measuring its linear expansion. This ramp was performed between 20 °C and 180 °C at a heating rate of 3 °C/min with an applied force of 0.01 N. During the test, the displacement of the sample was recorded in real time, allowing a thermal expansion curve to be plotted.

The coefficient of thermal expansion (*CTE*) was determined from the dimensional variations recorded during the thermal cycle. The formula used to calculate *CTE* is as follows:

(6) $$CTE=\frac{1}{L_{0}}\frac{\Delta L}{\Delta T}\;$$

where $L_{0}$ is the initial length of the sample, ΔL is the length variation during the analysis, and ΔT is the temperature change.

#### 2.2.5. Thermal Conductivity

To measure the thermal conductivity of PP, the Transient Plane Source (TPS) method was considered, in accordance with ISO 22007-2 [21]. The device used was the TPS 2200S (HotDisk AB, Göteborg, Sweden), which uses a sensor placed between two identical plates.

The analysis was carried out in “Standard Analysis” mode, allowing simultaneous measurement of temperature and heat flux in the sample. Several PP plates, measuring 100 × 80 × 2 mm, were thermoformed from Hifax 3080 granules for testing.

The sample was subjected to a transient heat source, generating a controlled thermal gradient. Thermal conductivity was then calculated based on the thermal response of the sample, according to the principles of the hot disk method [22]. This technique determines thermal conductivity by measuring the time required for the sample to reach a stable thermal state after heat application.

#### 2.2.6. Dynamic Mechanical Analysis

Dynamic mechanical analysis (DMA) is used to measure the shear storage modulus *G′*(T) as a function of temperature. This technique is particularly useful for obtaining information about the viscoelasticity of the material. To determine the Young’s modulus *E′*(T), it is necessary to know the evolution of the Poisson’s ratio *ν*(T) with temperature, as these quantities are related by the following equation:

(7) $$E^{\prime }=2G^{\prime }\left(1+\nu \right)$$

However, measuring the Poisson’s ratio ν(T) as a function of temperature is complex, as it requires analyzing both longitudinal and transverse thermal expansions of the sample. This type of analysis could be performed using digital image correlation, which was not feasible in our case. Therefore, the temperature-dependent Young’s modulus values were taken from the literature.

#### 2.2.7. Rheometry

To determine the healing degree $D_{h}(t)$, which evaluates the ability of polymer chains to diffuse and bond two printed layers, a dynamic rheometer (ARES-G2, TA Instruments, New Castle, DE, USA) in a parallel plate configuration was used to assess the viscoelastic behavior of the molten polymer. Tests were carried out on 25 mm diameter and 1 mm thick disks, across a temperature range representative of the FFF process (160 to 260 °C, with 10 °C intervals).

The methodology includes several steps. First, a strain sweep is performed to identify the optimal strain that keeps the material within the linear viscoelastic region. Next, a frequency sweep is conducted to obtain the storage modulus *G*′ (elastic behavior), the loss modulus *G*″ (viscous behavior), and the complex viscosity η*. The relaxation time $\tau_{w}(T)$ is extracted from the maxima of the Cole–Cole curves plotted from the elastic component of the complex viscosity (η″) vs. the viscous component of the complex viscosity (η′) and is used to model $D_{h}(t)$. This time is directly related to the molecular diffusion and reorganization of polymer chains. By incorporating $D_{h}(t)$ into the Arrhenius equation, the behavior of PP can be predicted as a function of temperature and interlayer contact time [23].

#### 2.2.8. PvT Behavior

The specific volume is a thermodynamic property that describes how a material’s volume changes with temperature and pressure. Direct measurement of this property is complex, requiring specialized equipment such as PvT cells, which simultaneously measure pressure and temperature while observing volume changes in the material. This analysis helps to characterize the thermal and physical behavior of the material under various conditions.

As direct measurements could not be performed in this study, the specific volume values were extrapolated from the literature data using similar PP materials.

#### 2.2.9. Emissivity

Since the emissivity of PP was not measured directly in this study, a value from the literature was used. Emissivity measurements are typically performed using instruments such as infrared spectrometers or thermal chambers, which analyze the radiation emitted by the material as a function of temperature and wavelength.

#### 2.2.10. Tensile Testing

Uniaxial tensile tests were carried out at room temperature to determine the stress and strain at break of the samples. The tests were performed using an MTS 2/M electromechanical testing system, MTS Systems Corporation, Eden Prairie, MN, USA equipped with a 1 kN load cell, at a crosshead speed of 10 mm/min, in accordance with ISO 527-2 [24]. This speed was selected to ensure fracture within a reasonable timeframe. A video extensometer was used to measure real strain. A minimum of five tensile specimens were tested for each reported value.

### 2.3. Optimization: Experimental Approach

#### 2.3.1. Design of Experiments

To establish a Taguchi experimental design, it is necessary to select the relevant factors and levels to test in order to assess the influence of the different parameters and identify the optimal levels. Although numerous studies exist on optimizing 3D printing parameters for materials such as PLA or ABS using the Taguchi method, it was hard to find any specific study on PP. Table 1 provides general guidelines on possible adjustments to the printing parameters and the variations in the main factors to be tested. The selection of main processing conditions was based primarily on the supplier data derived from the patent of Palo and Cavalieri [25] related to the 3D printing of the same PP filament. In this work, the main objective is to find a compromise between mechanical performance and printing time. Indeed, it is crucial that the printing time of orthoses does not exceed that of conventional methods, particularly in the context of humanitarian HI interventions, where time savings are essential to make the 3D printing method viable.

Five key parameters were selected to study their influence on the mechanical properties of the 3D-printed samples: extrusion temperature, printing speed, layer thickness, infill density, and infill pattern. Other parameters, such as bed temperature, retraction, and the number of perimeters, were fixed in order to reduce sources of variability. The selected parameters and their respective levels are presented in Table 2.

To optimize testing and reduce the number of experiments while still covering the design space, a Taguchi experimental design was employed using the MINITAB 16.1 software (Minitab LLC, State College, PA, USA). This method enables the extraction of maximum information with a minimal number of experiments, thus saving time and cost.

The selected design was based on an L25 orthogonal array, which is suitable for five factors at five levels each. This approach required only 25 experiments, compared to 3125 (5^5^) in a full factorial design. The experimental configuration is detailed in Table 3.

The samples were printed according to the conditions defined by this experimental matrix.

#### 2.3.2. Mechanical Property Evaluation Methods

To assess the mechanical properties of the 3D-printed parts, three-point bending and short-beam bending tests were carried out in compliance with ISO and ASTM standards.

##### Three-Point Bending Test

Three-point bending tests were conducted on printed specimens according to ISO 178 [26], with dimensions of 80 mm (length) × 10 mm (width) × 4 mm (thickness). The tests were performed using a universal testing machine (MTS 2/M system, MTS Systems Corporation, Eden Prairie, MN, USA).

The samples were printed based on the experimental plan, adjusting the printing parameters accordingly. To ensure result repeatability, three specimens were printed per Taguchi configuration. After printing, specimens were sealed in airtight plastic bags to prevent moisture absorption and contamination.

The tests were carried out with a load capacity of 10 kN. The bending fixture was set with a span length of 64 mm (16 times the specimen thickness). The crosshead displacement rate was maintained at 2 mm/min. At the end of each test, maximum stress and Young’s modulus were calculated.

##### Short-Beam Bending Test

Short-beam bending tests were carried out on specimens printed in accordance with ASTM D2344 [27]. Each specimen measured 24 mm × 8 mm × 4 mm. The tests were conducted on the MTS 2/M system, MTS Systems Corporation, Eden Prairie, MN, USA, with a dedicated short-span fixture.

The span length between supports was set to 20 mm, and a load cell of 1 kN was used. The crosshead speed was 2 mm/min. The maximum force was then recorded for analysis.

#### 2.3.3. Analysis Method

Optimization of the 3D printing parameters was carried out using the Taguchi method, based on the Signal-to-Noise (S/N) ratio. This ratio evaluates the effect of design factors on measured responses by distinguishing between a useful signal and random noise.

Two criteria were considered in this study:

Mechanical properties (Young’s modulus and maximum stress), for which a “larger-is-better” S/N ratio was used:

(8) $$\frac{S}{N}=-10log\left(\sum_{i=1}^{n}\frac{1}{y_{i}^{2}}\;\right)$$

Printing time, for which a “smaller-is-better” S/N ratio was applied:

(9) $$\frac{S}{N}=-10log\left(\sum_{i=1}^{n}y_{i}^{2}\;\right)\;\;$$

An analysis of variance (ANOVA) was then conducted for each response variable. This statistical method quantifies the influence of each factor on response variability and identifies the most significant parameters. Key outputs include the statistical significance of each factor (*p*-values), model lack of fit, agreement between adjusted and predicted R^2^, and the accuracy of the model’s predictions

Using this analysis, the 3D printing process was optimized by identifying influential parameters and prioritizing the responses based on a weighting scheme. Following optimization, an experimental validation was carried out to compare printed part performance with predicted values, ensuring the practical relevance of the optimized settings for enhancing both mechanical strength and printing efficiency.

#### 2.3.4. Mechanical Testing of the Orthosis

**Figure 1:**
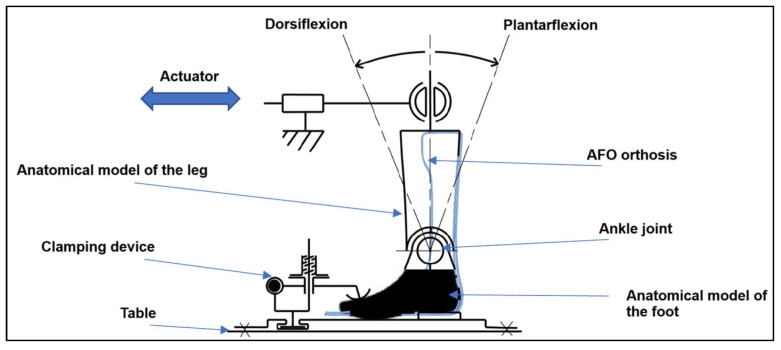
A schematic representation of the mechanical test bench for orthoses.

To evaluate the mechanical performance of the orthosis, a home-made biaxial testing machine with electromechanical actuators (horizontal configuration) was used (Figure 1). This setup enables uniaxial or biaxial tensile tests according to ISO 22675 [28]. The machine is equipped with four actuators, each with a maximum capacity of 2.5 kN and a stroke of 200 mm.

To adapt the setup for orthosis testing, a silicone anatomical leg model was added, onto which the orthosis was mounted (Figure 1). The anatomical model is connected via a ball joint to one actuator, reproducing a walking motion through a reciprocating actuator movement (Figure 1).

Several fixing points were used to secure the orthosis during testing. For foot fixation, the end of the orthosis was clamped using a metal plate and screws. For ankle fixation, a strap was used to stabilize the orthosis around the leg model. Finally, for tibial fixation, a second strap ensured proper and realistic attachment during testing (Figure 1).

In this study, quasi-static failure tests were performed, which are faster and provide quantitative insights into the strength and mechanical behavior of the orthosis under load. Indeed, according to Beckerman et al. [10], an AFO in dorsiflexion or plantarflexion positions will simulate normal walking (i.e., heel strike and foot unrolling). However, cyclic fatigue tests would have been performed to simulate real-world walking loads and evaluate long-term durability. These tests will be the subject of a future article.

#### 2.3.5. Optimization: Numerical Approach

In this study, Digimat-AM 2022.4 (MSC Software/Hexagon, Mont Saint-Guibert, Belgium) was used to simulate the 3D printing process of the orthoses. Specifically designed for additive manufacturing, this tool enables the prediction of residual stresses and warpage in parts printed with polymers and composites. The simulation process in Digimat-AM follows four main steps: model definition, virtual manufacturing, numerical simulation, and results analysis.

During the model definition phase, Digimat-AM offers three printing technologies: selective laser sintering (SLS), Fused Filament Fabrication (FFF), and fused deposition modeling (FDM). The FFF technology was selected for this study, as it corresponds to the process used by the WASP 4070 ZX printer. This printer features a build volume of 400 × 400 × 700 mm, a fixed platform, and a thermal control system that allows precise regulation of part cooling.

The analysis used in the simulation is warpage high fidelity, which accounts for detailed thermomechanical effects for improved deformation prediction.

##### Material Characterization

The polypropylene (PP) used for 3D printing was not available in the Digimat-MX database and had to be implemented manually. The material properties were defined using a combination of experimental data and literature values (Table 4).

##### Modeling and Simulation Conditions

The numerical simulation provided estimates of residual stresses and warpage in the printed orthoses. These insights are crucial for understanding deformation mechanisms and optimizing printing parameters to minimize dimensional deviations. For further details on how solvers work on Digimat-AM, readers could consult the pioneering work of Al Rashid et al. [30].

The orthosis geometry was imported into Digimat-AM as an STL file and meshed to ensure sufficient accuracy in the simulation.

During the virtual manufacturing phase, several parameters were defined to reproduce the actual printing conditions. The ambient temperature was set to 24 °C, and the chamber temperature was maintained at 30 °C. A convection coefficient of 0.015 mW/mm^2^·°C was applied to simulate heat transfer between the part and its surrounding environment.

A layer-by-layer discretization strategy was used to better capture thermomechanical effects throughout the printing process. A voxel size of 0.2 mm was used. Finally, the job was submitted for analysis, and the same procedure was adopted for all Design of Experiments (DOE) matrix runs. After the successful numerical calculation, the pertinent output results, such as deformation and residual stress, were imported for further processing.

## 3. Results and Discussion

### 3.1. Material Characterization

#### 3.1.1. Thermal Properties of PP Pellets and PP Filaments

The DSC thermograms for the TREED PLENE 5 filament and Hifax 3080 pellets are shown in Figures S1 and S2 of the Supplementary Materials. Due to the very low glass transition temperature (Tg) of polypropylene (PP), this transition is not visible on the thermograms. However, the melting and crystallization peaks are clearly defined. The melting temperature (Tm) is 167 °C for TREED PLENE 5 and 166 °C for Hifax 3080, while the crystallization temperature (Tc) is 121 °C for TREED and 123 °C for Hifax. In terms of thermal behavior, PP filament and PP pellets exhibit a similar signature.

The degree of crystallinity was also calculated using Equation (1). The results obtained were 27% for TREED PLENE 5 and 26% for Hifax 3080. These calculations assume a polypropylene mass fraction of 100%. According to supplier [25], PP pellet (HIFAX 3080 grade) is a heterophasic copolymer consisting of a PP matrix with Propylene/ethylene rubber nodules. These nodules account for 32% of the blend and contain about 17% ethylene, corresponding to approximately 5% ethylene in the total mass. This minor proportion was considered negligible and was thus excluded from the crystallinity rate calculation.

This relatively high crystallinity partly explains the shrinkage and associated warpage observed during the 3D printing of PP. It is therefore crucial to control the printing parameters. However, although shrinkage can be challenging, using a semi-crystalline polymer allows for better mechanical properties post-printing compared to an amorphous polymer.

The melting temperature, crystallization temperature, and crystallinity values of TREED PLENE 5 filament and Hifax 3080 grade are very close (Table S1). The latter was used to model the material in Digimat-AM software 2022.4.

#### 3.1.2. Specific Heat Capacity as a Function of Temperature

**Figure 2:**
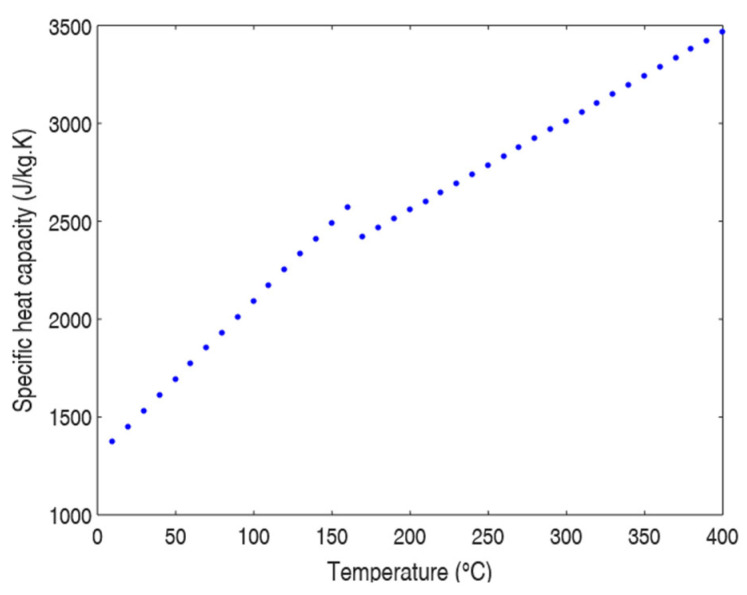
Specific heat capacity of PP as a function of temperature.

Figure 2 illustrates the evolution of specific heat capacity (Cp) with temperature obtained for both the crystalline and amorphous phases of polypropylene. A jump in Cp is observed as the melting temperature approaches.

#### 3.1.3. Crystallization Kinetics Data

Crystallization plays a key role in the 3D printing of PP, influencing the microstructure, mechanical properties, and shrinkage of the printed parts. To model this behavior, the Nakamura–Weibull model was used, which is particularly suited when cold crystallization is absent.

The Nakamura–Weibull model is based on a modified Avrami–Weibull growth law, describing the crystallization evolution over time and temperature.

The parameters were taken from the study of Yuesheng Yu et al. [31] and are listed below (Table 5):

The Hoffman–Lauritzen model can also be used to describe both isothermal and non-isothermal crystallization. It accounts for the temperature dependence of crystallization rates and is often used to characterize the behavior of semi-crystalline polypropylenes.

The constants used for this model, based on the study of Leandro Ariel Santoro et al. [32], are summarized below (Table 6):

#### 3.1.4. TGA Results

The results of the thermogravimetric analysis (TGA) are shown in Figure S3 of the Supplementary Materials. The onset temperature of degradation is around 370 °C, while the end of degradation occurs at approximately 470 °C.

The observed mass loss is $M_{L}$ = 98.82%. This indicates that the tested material does not contain mineral fillers, as the residue at the end of the heating process is less than 2%. Therefore, the potential impact of additives or mineral fillers on the 3D printing behavior of this material can be disregarded.

The thermal stability index value is TSI = 0.79. This value, being close to 1, indicates that the polypropylene (PP) is thermally stable.

#### 3.1.5. TMA Results

Dimensional changes in PP with temperature were measured using TMA, as shown in Figure S4 of the Supplementary Materials. The initial length L_0_ of the sample was 2061.7 µm. The relationship between dimensional change (ΔL) and temperature (T) was determined using a regression line, with the following equation: ΔL=0.532 T−17.425.

To extract the coefficient of thermal expansion (CTE), the slope of this curve is used, which is 0.532 µm/°C. Applying this value to Equation (5), we obtain an average CTE of $25.8\times 10^{-5}\;K^{-1}$ over the studied temperature range.

**Figure 3:**
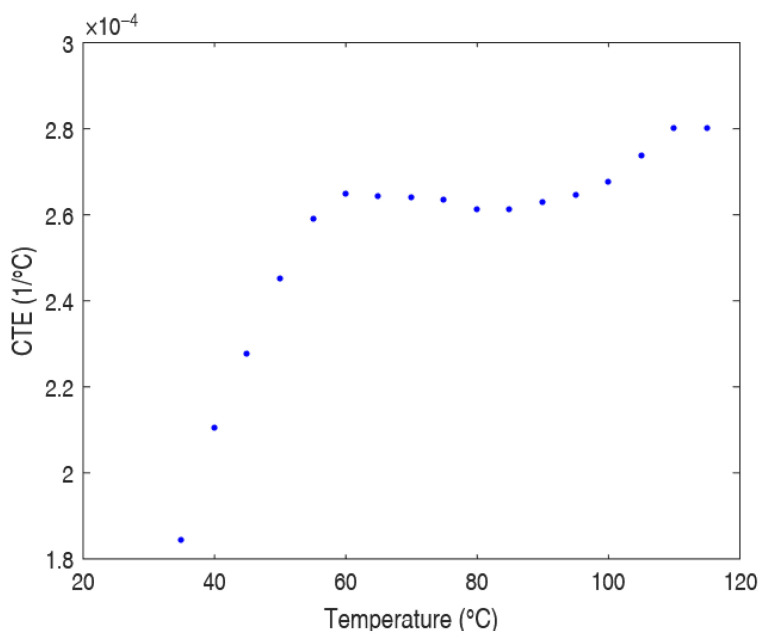
CTE of PP as a function of temperature.

Based on the obtained data, the variation in the CTE with temperature can also be assessed (Figure 3). By calculating the local CTE over several temperature intervals, it is possible to observe how it changes at different temperatures. The graph below shows the evolution of CTE with temperature, calculated from dimensional variations over specific intervals.

#### 3.1.6. Thermal Conductivity Data

Thermal conductivity values as a function of temperature at the solid and the melt states are depicted in Figure S5. Thermal conductivity measurements were conducted both on solid PP (from 20 °C to 150 °C) and amorphous PP (from 170 °C to 200 °C). These measurements showed that the thermal conductivity of PP remains relatively constant over the studied temperature range.

#### 3.1.7. DMA Data

For the evolution of the Young’s modulus with temperature, experimental data from the article of Coppola et al. [33] were used (Figure S6). The authors studied isotactic PP/EPR blends with 0%, 10%, and 20% EPR mass fractions. Although the exact composition of our grade (70/30) is not directly covered in the study, the available data are still relevant for our estimations.

The samples in the study of Coppola et al. [33] were crystallized at 20 °C, 80 °C, and 126 °C, and then mechanically tested between 20 °C and 120 °C. We selected the data corresponding to crystallization at 20 °C, as this temperature best reflects the rapid cooling conditions encountered in FFF 3D printing.

To estimate the Young’s modulus for our 70/30 composition, we performed a linear interpolation between the values of the 80/20 and 90/10 blends.

#### 3.1.8. PvT Data

The experimental data for the specific volume of PP as a function of temperature and pressure (PVT) were extracted from the study of Paul Zoller [34] (Figure S7). These measurements were performed on an isotactic PP (iPP, Vestolen P 6200 grade) over a temperature range of 30 °C to 297 °C and pressures up to 200 MPa. The results for solid and molten PP were fitted using the equation of state based on the Tait equation [34], allowing reproduction of the specific volume values with an accuracy of less than 0.001 cm^3^/g.

#### 3.1.9. Emissivity

According to available data [35], the emissivity of PP is 0.94 within the wavelength range of 2 to 5.6 µm. An emissivity close to 1 indicates that the material behaves as a good thermal emitter, meaning it efficiently absorbs and re-emits energy as infrared radiation. This value was used for the thermal analysis of the material.

#### 3.1.10. Tensile Testing

The tensile stress–strain curve of PP is presented in Figure S8 (see the Supplementary Materials). The experimental results were compared with the values provided in the material’s datasheet. Table 7 summarizes the comparison. The experimental results are consistent with the expected values.

### 3.2. Results of the Design of Experiments

#### 3.2.1. Normal Probability of Residuals

**Figure 4:**
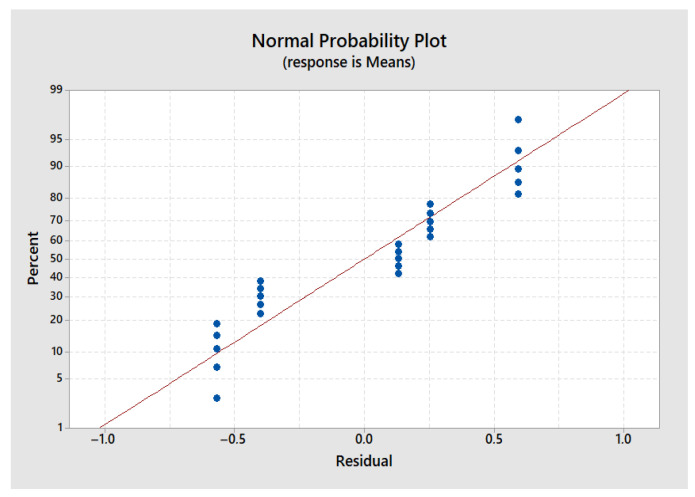
Normal probability plot—maximum stress.

Figure 4, representing the normal probability of residuals, shows that the data points are generally aligned along the normality line, indicating that the residuals follow a normal distribution. This suggests that the statistical assumptions underlying the ANOVA are met, allowing for a reliable analysis of the effects of printing parameters on the residual stress.

**Figure 5:**
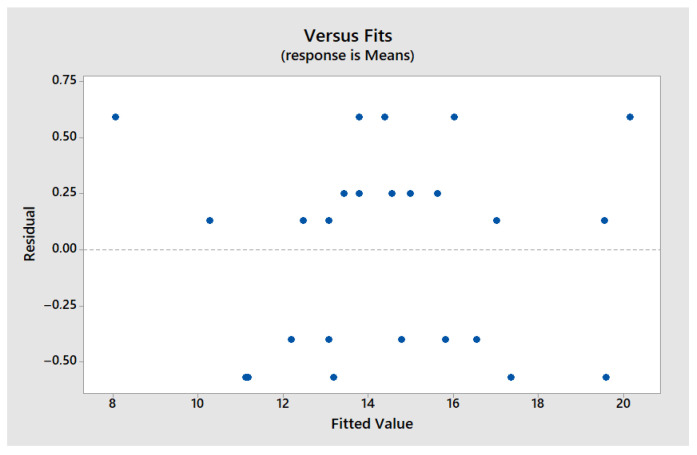
Residuals vs. fit—maximum stress.

The second graph (Figure 5), which plots the residuals against the fitted values, reveals no structured pattern, indicating a homogeneous dispersion of errors. This random distribution supports the assumption of constant variance, which is essential for the correct interpretation of ANOVA results and S/N ratios. Thus, the analyses can be conducted without risk of statistical bias.

These two graphs concern the maximum stress, but similar observations were made for the other measured responses: flexural modulus, printing time, and ILSS.

Table 8 presents the experimental plan that varies five process parameters along with the results of the tests conducted according to this configuration. The test results show that specimen no. 8 achieved the best performance in three-point bending, with a flexural modulus of 818 MPa and a maximum stress of 21 MPa. Specimen no. 19 stood out in the short-span bending test, displaying the best ILSS value (2.85 MPa) and the second-best result in three-point bending. These results indicate that specific parameter combinations lead to a significant improvement in the mechanical properties of the printed parts.

#### 3.2.2. Three-Point Bending Test

##### Effect of Parameters on Maximum Stress

**Figure 6:**
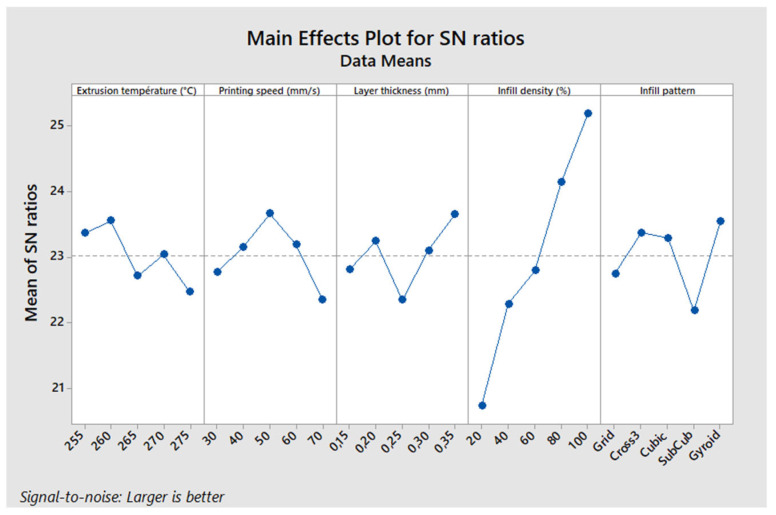
Main effects plot—maximum stress.

Figure 6 illustrates the main effects of the parameters on the maximum stress in a three-point bending test. The “larger is better” approach was used to analyze the Signal-to-Noise (S/N) ratio. This analysis shows that the optimal configuration for maximizing the maximum stress corresponds to an extrusion temperature of 260 °C, a printing speed of 50 mm/s, a layer thickness of 0.35 mm, an infill density of 100%, and a Gyroid infill pattern. This specific combination of parameters, corresponding to levels A2, B3, C5, D5, and E5, yields the highest maximum stress.

Table 9 presents the ANOVA results and the contribution of each factor to the variation in maximum stress. The contribution percentage was calculated using the following formula:

(10) $$\%\;of\;contribution\;=\;\frac{SeqSS}{TotalSS}\times 100$$

The results show that the infill density is the most influential parameter, contributing 73.2% to the optimization of maximum stress. It is followed by the infill pattern (7.79%), printing speed (6%), layer thickness (5.91%), and finally, extrusion temperature, which has a more limited effect (4.99%). The predominant impact of the infill density is explained by its direct influence on the density and load distribution within the printed part, thus affecting its mechanical strength.

**Figure 7:**
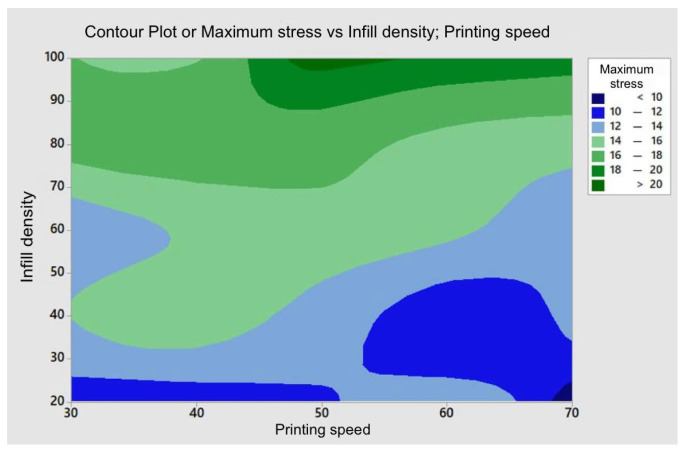
Contour plot—maximum stress as a function of infill density and printing speed.

A contour plot (Figure 7) was created to examine the combined influence of the infill density and printing speed, as these two parameters are the most influential (the infill pattern cannot be represented this way). This analysis shows that to maximize the maximum stress, the printing speed should be around 50 mm/s, and the infill density should be higher than 95%.

##### Effect of Parameters on Flexural Modulus

**Figure 8:**
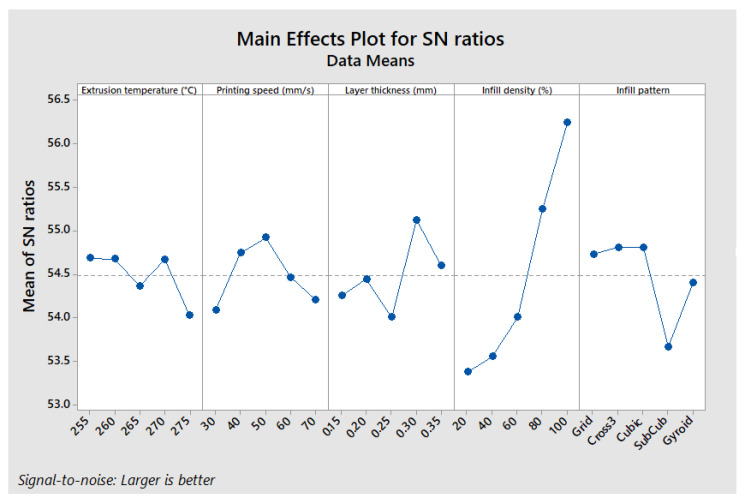
Main effects plot—flexural modulus.

The same analysis was conducted for the flexural modulus. According to the results, the optimal configuration to maximize this mechanical property corresponds to an extrusion temperature of 260 °C or 270 °C, a printing speed of 50 mm/s, a layer thickness of 0.3 mm, an infill density of 100%, and either a Cubic or Cross 3D infill pattern (both performing similarly). This combination, corresponding to levels A2-4, B3, C4, D5, and E2-3, yields the highest flexural modulus values (Figure 8).

Table 10 presents the ANOVA results for the flexural modulus and highlights the contribution of each factor. As before, the infill density emerges as the dominant parameter, contributing 62.86% to the maximization of the flexural modulus. It is followed by the infill pattern (9.91%), layer thickness (7.43%), printing speed (5.18%), and finally, extrusion temperature (3.54%).

##### Effect of Parameters on Printing Time

The evaluation of printing time was based solely on estimates provided by the Ultimaker Cura slicing software. However, it is important to note that the estimated time does not always match the actual printing time and can be underestimated by up to 30%. It was not feasible to continuously monitor each print to measure the actual time, which is why Cura values were used in this analysis.

**Figure 9:**
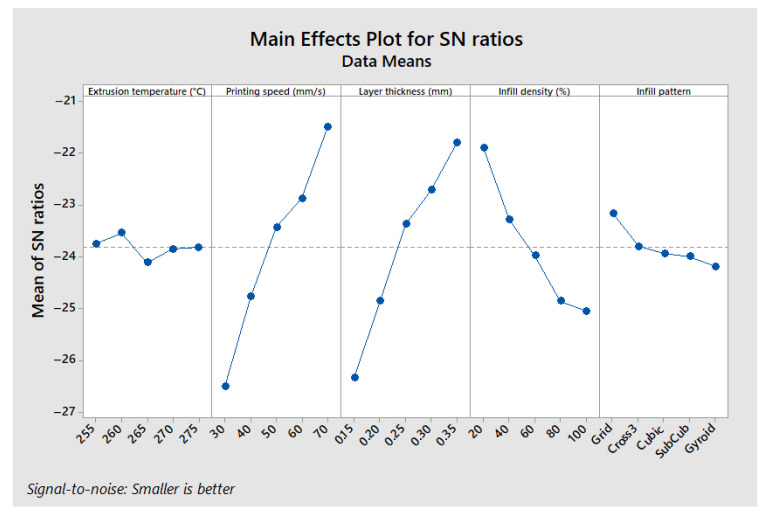
Main effects plot—printing time.

Figure 9 illustrates the main effects of the parameters on the printing time for the flexural test specimens. Unlike previous analyses where mechanical performance was maximized, the goal here is to minimize printing time. Therefore, the “smaller is better” approach was used when calculating the S/N ratio. The observed trends are consistent: an extrusion temperature of 260 °C, a printing speed of 70 mm/s, a layer thickness of 0.35 mm, an infill density of 20%, and a Grid infill pattern (configuration A2, B5, C5, D1, and E1) result in the shortest printing time.

However, these results cannot be interpreted in isolation. While this configuration minimizes printing time, it does not necessarily ensure the best mechanical properties. Thus, it is essential to find a compromise between reducing printing time and optimizing mechanical performance.

Table 11 shows the ANOVA results for printing time, highlighting the contribution of each factor. Unlike mechanical performance, here the printing speed has the most influence on printing time, with a contribution of 41.4%, followed by layer thickness (36.6%) and infill density (18.7%). In contrast, the infill pattern and extrusion temperature have negligible effects, contributing only 1.7% and 0.5%, respectively. These results confirm that increasing the printing speed and layer thickness are the most effective levers to reduce printing time, whereas infill density and pattern are more important for mechanical performance.

#### 3.2.3. Short-Beam Bending Test

A complementary study was conducted on close-range bending tests, specifically on the Interlaminar Shear Strength (ILSS). This property is essential to evaluate the cohesion between printed layers and their ability to withstand mechanical stresses.

**Figure 10:**
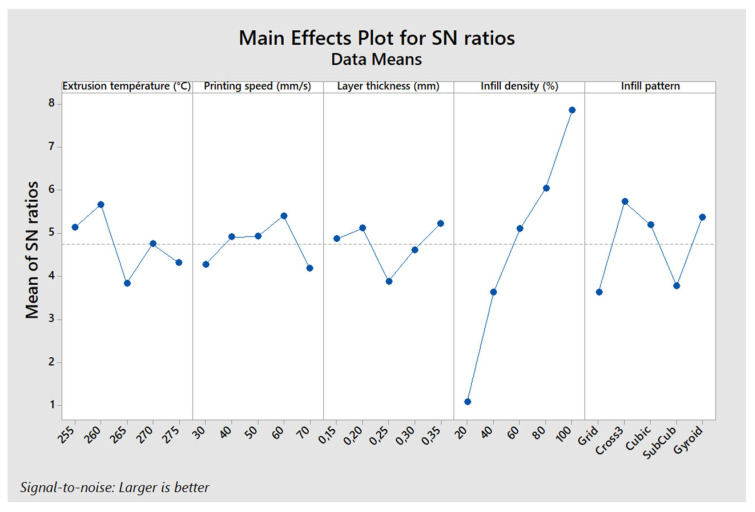
Main effects plot—ILSS.

The results indicate that the optimal configuration for maximizing ILSS is as follows: extrusion temperature of 260 °C, printing speed of 60 mm/s, layer thickness of 0.35 mm, infill density of 100%, and a Cross 3D infill pattern (A2, B4, C5, D5, and E3). This configuration differs slightly from the one identified for maximum stress in the three-point bending test (A2, B3, C5, D5, and E5), although overall trends remain similar (Figure 10).

The ANOVA results presented in Table 12 show that the infill density is once again the most influential factor, contributing 75.2% to the optimization of ILSS (Interlaminar Shear Strength). The infill pattern follows with 10.7%, while the extrusion temperature has a more moderate effect (5.8%). In contrast, the printing speed (2.9%) and the layer thickness (3.3%) have a relatively low impact.

These findings confirm that increasing the infill density and choosing an appropriate infill pattern are the most critical factors for enhancing interlaminar cohesion. The influence of other parameters remains secondary, which is consistent with previous observations regarding the mechanical strength of printed parts.

#### 3.2.4. Optimal Parameters

To confirm the optimal configurations and validate the design of experiments (DOE), mechanical tests were conducted using the parameters that maximize mechanical properties. The experimental results were then compared to the values predicted by the numerical model, as shown in Table 13.

To assess the deviation between the predicted and actual values, the following formula was used to calculate the relative error with respect to the predicted value:

(11) $$\%\;Error=\frac{\left|predicted\;value-experimental\;value\right|}{predicted\;value}\times 100$$

The error between experimental values and those predicted by the model remains generally acceptable for maximum stress and flexural modulus, with a deviation under 15%. However, a significant discrepancy was observed for ILSS, with a deviation exceeding 32%. This difference is likely due to the model not being fully adapted to the nature of PP, which is a relatively flexible material (i.e., deformation of the PP matrix during flexural test reduces the stress transfer at the interfaces between the 3D-printed layers), unlike the rigid composites for which ILSS is typically used as a performance criterion. In addition, possible causes such as the limitations of the isotropic material model in Digimat-AM and the challenges of simulating PP’s viscoelastic behavior contribute to the observed gap between experimental ILSS and prediction values.

The following configurations were identified:

- Configuration 1: ILSS optimization (A2, B4, C5, D5, and E3)
- Configuration 2: Flexural modulus optimization (A4, B3, C4, D5, and E2)
- Configuration 3: Maximum stress optimization (A2, B3, C5, D5, and E5)

These configurations were then tested on orthoses to assess whether they actually improve mechanical performance.

#### 3.2.5. Tests on Orthoses

Finally, orthoses were printed with different configurations to validate the improvement in mechanical properties as well as surface finishes. Further analyses will be carried out using Digimat-AM, allowing a more in-depth study of printing parameter variations.

The printed orthoses and their associated parameters are shown in Table 14.

**Figure 11:**
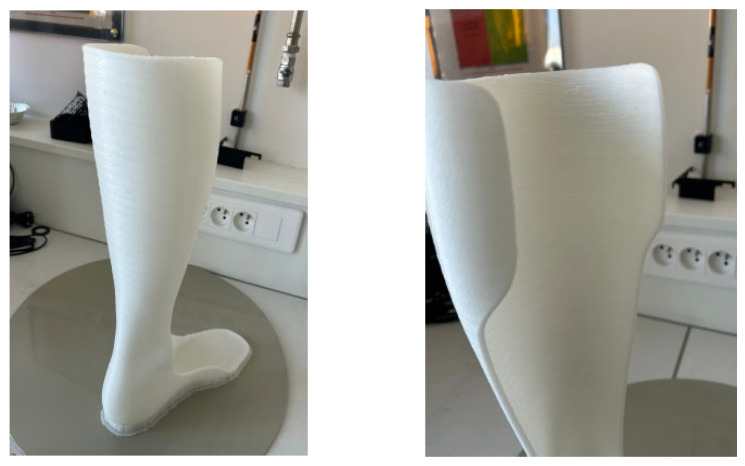
Printed adult AFO orthosis 2.

Observation of the printed orthoses revealed several points. First, orthosis 1 showed the best surface finish (Figure 11), while orthosis 2, printed with a 0.35 mm layer height, showed excessive material deposition in the form of residual strings (stringing), indicating a need for retraction parameter optimization to reduce this phenomenon.

Regarding the printing supports, a Grid infill (10 to 15%) was used. However, removing them proved particularly difficult and even led to the breakage of the first orthosis during detachment. A potential solution would be to remove the supports immediately after printing, while the part is still warm, to facilitate removal. Alternatively, reheating the supports post-printing could be attempted, though this approach carries a risk of thermal deformation of the orthosis.

Finally, to achieve an optimal surface finish, post-processing of the bottom of the orthosis is necessary. Progressive sanding, starting with 150–220 grit to reduce ridges and imperfections, followed by finer sanding with 320–340 grit, could improve the surface appearance. A rotary sander could be used for this. Unfortunately, the absence of appropriate equipment did not allow post-processing to be performed in this study.

The five orthoses were then tested using a biaxial test device to evaluate their mechanical performance. The results are shown below (Table 15):

In the test results, a notable variation in mechanical performance was observed based on the fabrication parameters, particularly layer height, infill pattern, and infill density. These parameters directly influence the strength and durability of the orthoses.

**Figure 12:**
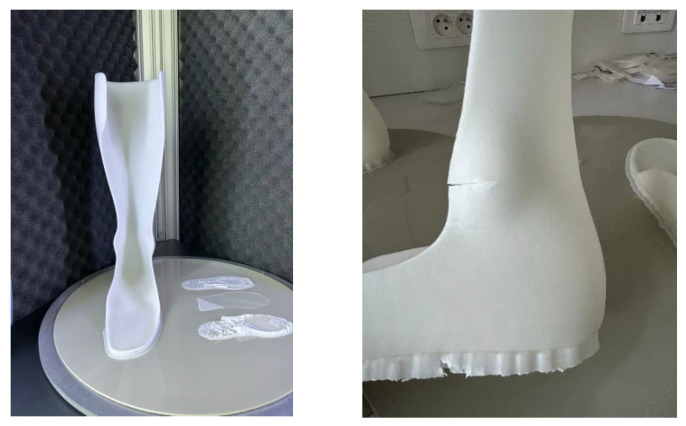
Printed adult AFO orthosis 1.

**Figure 13:**
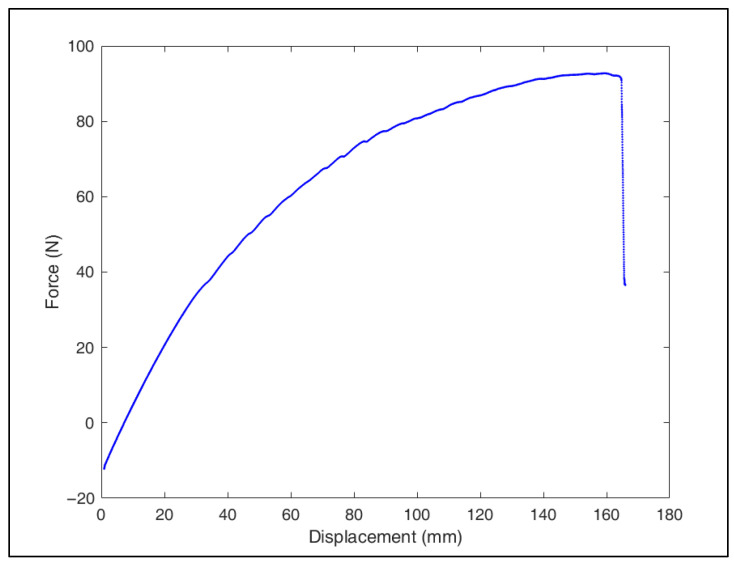
Tensile testing curve of the orthosis 4.

Orthoses with thicker layer heights, such as orthoses 2 and 5, exhibited more brittle and sudden failures. This suggests that thicker layers may create weak points in the structure, leading to quicker failure under pressure and negatively affecting orthosis durability. Additionally, the surface finish of these orthoses was poorer, with printing defects as shown in Figure 12. In contrast, orthoses with thinner layers and higher infill density, such as orthoses 3 and 4, demonstrated more gradual failures. Thus, it is recommended not to exceed a 0.25 mm layer height to maintain good surface quality and ensure sufficient mechanical strength (Figure 13).

The infill pattern also plays a crucial role. The Cubic pattern used in orthoses 3 and 4 provides better strength and flexibility before failure, which is essential for durability and comfort. On the other hand, Grid or Gyroid patterns offer less optimal mechanical performance, particularly in terms of breaking force, although they may reduce printing time.

Printing time is a key factor, especially in contexts where rapid production is essential, such as in humanitarian missions. Orthoses with high infill densities (like orthosis 1) take up to 34 h to print, which is too long in urgent situations. However, orthosis 4, with an 85% infill density, offers comparable performance while reducing print time to 22 h, representing an optimal compromise. Indeed, lower infill densities might be sufficient to maintain the required strength while further reducing print time. Moreover, as highlighted by Peng et al. [36], orthoses experience horizontal displacements not exceeding 25 mm in normal use, while the tested orthoses failed after displacements exceeding 100 mm, providing a safety factor of about 4, which shows that current performance is more than adequate for patient needs.

In conclusion, orthosis 4 is the optimal configuration, offering a perfect compromise between mechanical performance (second best after orthosis 3) and a reduced printing time of 22 h. This configuration is particularly suitable for environments where rapid production is crucial, such as in humanitarian missions, where efficiency and reduced print time are essential without compromising performance.

### 3.3. Numerical Simulation Results

Since polypropylene (PP) is not included in the default Digimat database, experimental procedures were carried out to determine its thermal and mechanical properties. The data obtained from these tests (Section 1), as well as data from the literature, were used to define the material’s behavior, including parameters such as the variation in Young’s modulus with temperature, thermal expansion, thermal conductivity, and other properties relevant to 3D printing. The characteristics of PP were integrated into Digimat-MX, allowing the simulation of its behavior during the FFF printing process.

#### 3.3.1. Optimal Orthosis Configurations

The results obtained are presented in Table 16 below:

The results show that orthosis 3 exhibits the lowest warpage (5.41 mm), indicating better dimensional stability. Orthosis 4, on the other hand, has the lowest von Mises stress (3.94 MPa), suggesting better resistance to plastic deformation. However, the stress levels remain below the yield strength of PP (≈15 MPa) for all configurations except the first, indicating mechanical compliance with the material limits.

**Figure 14:**
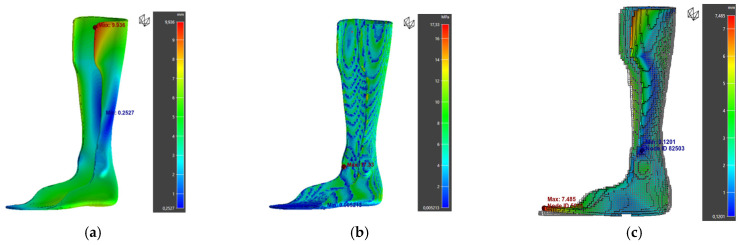
Simulation results of orthosis 1: (**a**) warpage, (**b**) von Mises Stress, (**c**) deviation from the initial part.

The warpage zones were mainly located at the calf tab and toe regions (Figure 14). These deformations, which are hard to detect visually, could be confirmed by a comparative 3D scan between the printed part and the digital model.

**Figure 15:**
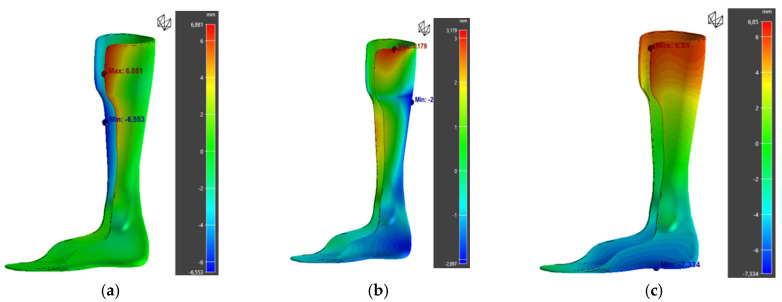
Warpage along the X (**a**), Y (**b**), and Z (**c**) axes.

Furthermore, analysis of warpage along the X, Y, and Z axes shows that deformations are mainly concentrated along the X and Z directions (Figure 15). This trend can be attributed to the material’s anisotropy and the stresses generated by the layer-by-layer deposition process.

#### 3.3.2. Influence of Print Orientation

In this study, three print orientations were considered: 0° (flat), 45°, and 90°. However, the 0° orientation could not be tested due to the orthosis dimensions, which did not allow flat placement on the build plate. Therefore, only the 45° and 90° orientations were evaluated through numerical simulations.

According to the literature [37], printing at 45° could enhance the mechanical performance of orthoses, as the applied forces during use are mainly horizontal. A 45° orientation theoretically enables better stress distribution across the printed structure, improving mechanical resistance.

**Figure 16:**
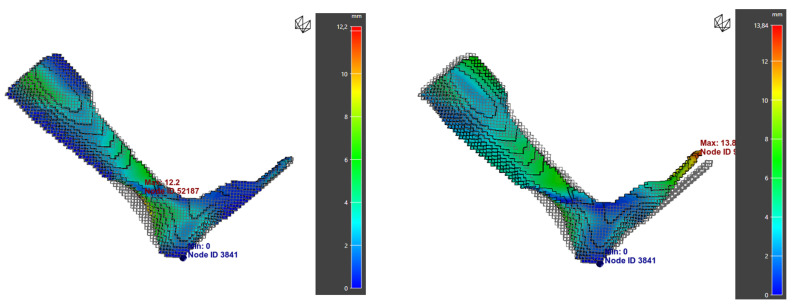
Simulation results for orthosis 3 at 45°: warpage and deviation from the initial part.

However, the results show a significant increase in warpage for the 45° orientation (33.61 mm compared to 5.41 mm for the 90° orientation), along with a slight increase in von Mises stress (Figure 16). These results suggest that, despite the literature recommendations, the 45° orientation is unsuitable in this specific case. The increased warpage (Table 17) could be linked to a higher accumulation of internal stresses during cooling, as well as a greater need for print supports, which complicates the process and compromises part stability.

Therefore, the 90° orientation appears to be the most suitable, as it minimizes deformation while maintaining acceptable mechanical stress. These findings highlight the importance of experimentally verifying theoretical recommendations, which can vary depending on the specific geometry and manufacturing conditions.

#### 3.3.3. Warpage Compensation

A key feature of Digimat-AM is the ability to generate a compensated model to reduce warpage. This process involves creating a modified STL file based on predicted deformations, allowing for material shrinkage to be anticipated.

**Figure 17:**
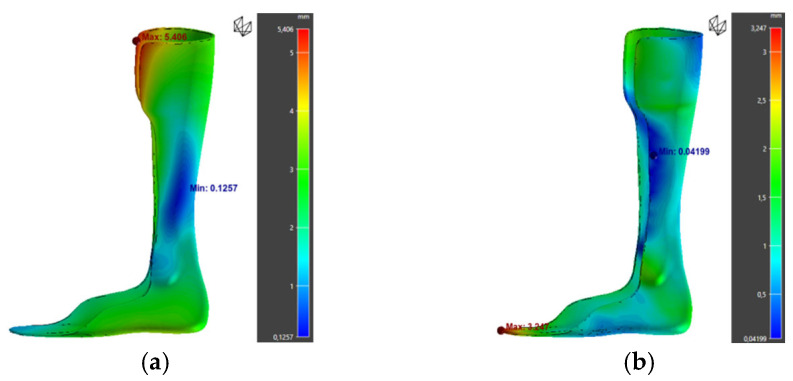
Simulation results for orthosis 3: (**a**) normal warpage; (**b**) warpage after compensation.

Applying this method to orthosis 3 yielded promising results: warpage was reduced from 5.41 mm to 3.25 mm, although the deformation shifted toward the toe areas (Figure 17). These results suggest that compensation significantly improves dimensional stability (Table 18). However, experimental validation through printing and 3D scanning is necessary to confirm its effectiveness under real conditions.

#### 3.3.4. Optimal Configuration

Determining an optimal configuration remains a trade-off between several criteria. Orthosis 3 offers the best balance in terms of dimensional stability, while orthosis 4 significantly reduces mechanical stress, notably thanks to an 85% infill density.

For further optimization, a Taguchi design of experiments directly applied to the orthoses could be considered. By varying infill density and layer thickness simultaneously, their respective influences could be identified, and the most efficient configuration determined.

## 4. Conclusions

This study enabled the optimization of 3D printing parameters for the fabrication of polypropylene (PP) orthoses, based on an experimental approach using the Taguchi method. The analysis of the results highlighted the most influential parameters on mechanical performance and printing time, particularly the infill density and pattern, which played a key role in the final strength of the printed parts. This approach made it possible to identify several optimal configurations, with parameter settings tailored to maximize both the maximum stress and the flexural modulus.

Among these, orthosis 4 was identified as the best configuration, with an 85% infill density, 0.2 mm layer thickness, Cubic infill pattern, and a printing speed of 50 mm/s. This configuration proved promising, offering a good balance between mechanical strength and reduced printing time. In addition, all tested orthoses met the ISO 178 standard for flexural performance, validating their feasibility for functional use.

However, to fully comply with the international ISO 22675 standard, fatigue testing would be required to assess the durability of the orthoses under cyclic loading. These tests would help ensure their long-term reliability under real-life usage conditions.

From a numerical standpoint, this study revealed certain limitations in predicting warpage and mechanical properties, due to the semi-crystalline nature of PP and interlayer interactions. Integrating a thermoviscoelastic (TVE) modeling approach would be an interesting perspective to improve the accuracy of these predictions and enhance the reliability of simulations within Digimat. Such a model would better account for temperature and creep effects on printed part deformation, thereby reducing the gap between simulations and experimental results.

In addition, the assumptions made in the simulation model (e.g., isotropy), the relatively small number of test samples, and the lack of long-term validation through real-world use shall be considered. The clinical significance of the optimized configurations should also be emphasized. For instance, orthosis 4 demonstrated a reasonable trade-off between mechanical strength and print time (22 h), making it more feasible for deployment in field conditions. Therefore, orthosis 4′s performance-to-time ratio indicates its suitability for rapid response scenarios in humanitarian contexts.

In conclusion, this study represents a step forward toward the optimized manufacturing of 3D-printed orthoses. Future improvements lie in experimental fatigue validation and the refinement of numerical models to ensure reliable and durable mechanical performance of PP-based orthoses.

## Figures and Tables

**Table 1:** Print settings used for FFF 3D printing of PP.

Variables	Values
Infill Density/Pattern	100%/concentric
Layer Width/Thickness	0.6 mm/0.2 mm
Number of Outlines	2
Build Plate/Nozzle Temperature	23/260 °C
Infill/External/Initial Speed Build Plate Adhesion	50/30/20 mm/min PP plate + raft

**Table 2:** Printing parameters and their variation levels.

Variables	Units	1	2	3	4	5	Symbol
**Extrusion temperature**	°C	255	260	265	270	275	A
**Printing speed**	mm/s	30	40	50	60	70	B
**Layer thickness**	mm	0.15	0.20	0.25	0.30	0.35	C
**Infill density**	%	20	40	60	80	100	D
**Infill pattern**		Grid	Cross 3D	Cubic	Cubic subdivision	Gyroid	E

**Table 3:** L25 orthogonal array used for the experimental study.

Test	Variables				
	A	B	C	D	E
**1**	255	30	0.15	20	Grid
**2**	255	40	0.2	40	Cross 3D
**3**	255	50	0.25	60	Cubic
**4**	255	60	0.3	80	Cubic subdivision
**5**	255	70	0.35	100	Gyroid
**6**	260	30	0.2	60	Cubic subdivision
**7**	260	40	0.25	80	Gyroid
**8**	260	50	0.3	100	Grid
**9**	260	60	0.35	20	Cross 3D
**10**	260	70	0.15	40	Cubic
**11**	265	30	0.25	100	Cross 3D
**12**	265	40	0.3	20	Cubic
**13**	265	50	0.35	40	Cubic subdivision
**14**	265	60	0.15	60	Gyroid
**15**	265	70	0.2	80	Grid
**16**	270	30	0.3	40	Gyroid
**17**	270	40	0.35	60	Grid
**18**	270	50	0.15	80	Cross 3D
**19**	270	60	0.2	100	Cubic
**20**	270	70	0.25	20	Cubic subdivision
**21**	275	30	0.35	80	Cubic
**22**	275	40	0.15	100	Cubic subdivision
**23**	275	50	0.2	20	Gyroid
**24**	275	60	0.25	40	Grid
**25**	275	70	0.3	60	Cross 3D

**Table 4:** Thermal and mechanical characterization test methods and standards of PP for numerical modeling.

Property	Test Method	Standard
Thermal transition	DSC	–
Heat capacity (Cp) vs. temperature	DSC (heat flow)	ASTM E-1269-11 [29]
Thermal stability index (TSI)	TGA	–
Coefficient of thermal expansion (CTE) vs. temperature	TMA	ASTM E831-14/ISO 11359-2
Thermal conductivity vs. temperature	TPS (Transient Plane Source)	ASTM E1530-19
Specific volume vs. temperature	PvT	ISO 17744:2004
Young’s modulus vs. temperature	DMA	ASTM D5279/ISO 6721/ASTM D4065
Crystallization kinetics	Isothermal DSC (Nakamura–Weibull model)	ASTM 1269-11

**Table 5:** Parameters used for the Nakamura–Weibull model.

Parameter	Value
Shape parameter	2.5
Scale parameter	148
Magnitude parameter	2.8
Avrami index	2.93

**Table 6:** Parameters used for the Hoffman–Lauritzen model.

Symbol	Unit	Value
$T_{g}$	K	273.15
$T_{m}^{0}$	K	451
n	−	2.24
$K_{g}$	$K^{2}$	$4.1\times 10^{5}$
${\left(1/t_{1/2}\right)}_{0}$	$s^{-1}$	$3.0\times 10^{9}$
$t_{m}$	${s\cdot K}^{a}$	$1.7\times 10^{14}$
a	−	7.85
$U^{*}$	J/mol	6284

**Table 7:** Comparison of tensile test results with supplier datasheet values.

Property	Elastic Stress (MPa)	Ultimate Stress (MPa)	Strain at Elastic Limit (%)	Strain at Break (%)
**Datasheet**	17	15	6	500
**Experimental results**	17.6	17.4	-	452

**Table 8:** Table of the experimental results of PP specimens.

Test	Variables					Three-Point Bending Responses			Short-Beam Bending Responses	
A		B	C	D	E	Flexural Modulus (MPa)	Maximum Stress (MPa)	Time (min)	ILSS (MPa)	Time (min)
**1**	255	30	0.15	20	Grid	477 ± 22	10 ± 0	19.7	0.98 ± 0.03	7.3
**2**	255	40	0.2	40	Cross 3D	549 ± 21	15 ± 0.3	18.3	1.93 ± 0.01	5.7
**3**	255	50	0.25	60	Cubic	535 ± 26	15 ± 0.2	14.7	1.96 ± 0.05	4.3
**4**	255	60	0.3	80	Sub. cubic	567 ± 45	15 ± 0.1	14.0	1.92 ± 0.06	3.7
**5**	255	70	0.35	100	Gyroid	590 ± 44	19 ± 0.6	11.7	2.71 ± 0.04	3.3
**6**	260	30	0.2	60	Sub. cubic	405 ± 5	13 ± 0.2	25.0	1.74 ± 0.03	7.0
**7**	260	40	0.25	80	Gyroid	597 ± 1	17 ± 0.5	17.7	2.16 ± 0.02	5.0
**8**	260	50	0.3	100	Grid	818 ± 26	21 ± 0.6	13.7	2.48 ± 0.01	4.0
**9**	260	60	0.35	20	Cross 3D	504 ± 16	14 ± 0.3	8.7	1.72 ± 0.02	3.3
**10**	260	70	0.15	40	Cubic	467 ± 6	13 ± 0.3	14.7	1.63 ± 0.01	6.0
**11**	265	30	0.25	100	Cross 3D	589 ± 5	16 ± 0.2	24.0	2.06 ± 0.04	6.7
**12**	265	40	0.3	20	Cubic	484 ± 17	11 ± 0.4	13.3	1.06 ± 0.02	4.3
**13**	265	50	0.35	40	Sub. cubic	476 ± 7.1	13 ± 0.2	11.0	1.29 ± 0.04	3.7
**14**	265	60	0.15	60	Gyroid	505 ± 27	14 ± 0.5	20.7	1.95 ± 0.07	6.3
**15**	265	70	0.2	80	Grid	571 ± 39	15 ± 0.2	14.7	1.66 ± 0.13	4.7
**16**	270	30	0.3	40	Gyroid	500 ± 8	14 ± 0.3	18.7	1.63 ± 0.04	5.3
**17**	270	40	0.35	60	Grid	539 ± 17	14 ± 0.2	13.0	1.65 ± 0.02	4.0
**18**	270	50	0.15	80	Cross 3D	573 ± 10	17 ± 0.3	23.3	2.29 ± 0.08	6.3
**19**	270	60	0.2	100	Cubic	714 ± 15	20 ± 0.5	17.3	2.85 ± 0.26	5.0
**20**	270	70	0.25	20	Sub. cubic	421 ± 16	9 ± 0.1	9.3	0.88 ± 0.02	3.7
**21**	275	30	0.35	80	Cubic	584 ± 19	17 ± 0.2	19.3	2.06 ± 0.02	5.3
**22**	275	40	0.15	100	Sub. cubic	566 ± 20	16 ± 0.3	27.7	2.32 ± 0.03	7.3
**23**	275	50	0.2	20	Gyroid	450 ± 8	12 ± 0.1	14.0	1.19 ± 0.03	5.0
**24**	275	60	0.25	40	Grid	400 ± 8	11 ± 0.4	12.0	1.22 ± 0.04	4.3
**25**	275	70	0.3	60	Cross 3D	538 ± 20	13 ± 1.5	10.0	1.73 ± 0.15	3.7

**Table 9:** ANOVA data for maximum stress.

Source	DF	Seq SS	Adj SS	Adj MS	F	p	% of Contribution
**Temperature**	4	4.03	4.03	1.01	2.42	0.21	4.99
**Printing speed**	4	4.84	4.84	1.21	2.91	0.16	6
**Layer thickness**	4	4.77	4.77	1.19	2.86	0.17	5.91
**Infill density**	4	59.09	59.09	14.77	35.5	0	73.23
**Infill pattern**	4	6.29	6.29	1.57	3.77	0.11	7.79
**Residual error**	4	1.67	1.67	0.42			
**Total**	24	80.68					

**Table 10:** ANOVA for flexural modulus.

Source	DF	Seq SS	Adj SS	Adj MS	F	p	% of Contribution
**Temperature**	4	1.69	1.69	0.42	0.32	0.853	3.54
**Printing speed**	4	2.47	2.47	0.62	0.47	0.76	5.18
**Layer thickness**	4	3.54	3.54	0.89	0.67	0.646	7.43
**Infill density**	4	29.97	29.97	7.49	5.67	0.061	62.86
**Infill pattern**	4	4.72	4.72	1.18	0.89	0.542	9.91
**Residual error**	4	5.28	5.28	1.32			
**Total**	24	47.68					

**Table 11:** ANOVA for printing time.

Source	DF	Seq SS	Adj SS	Adj MS	F	p	% of Contribution
**Temperature**	4	0.85	0.85	0.21	0.41	0.793	0.48
**Printing speed**	4	73.52	73.52	18.38	35.8	0.002	41.4
**Layer thickness**	4	64.93	64.93	16.23	31.61	0.003	36.56
**Infill pattern**	4	33.14	33.14	8.29	16.14	0.01	18.66
**Infill Pattern**	4	3.09	3.09	0.77	1.5	0.351	1.74
**Residual error**	4	2.05	2.05	0.51			
**Total**	24	177.6					

**Table 12:** ANOVA results for ILSS.

Source	DF	Seq SS	Adj SS	Adj MS	F	p	% of Contribution
**Temperature**	4	10.02	10.02	2.51	3.75	0.115	5.76
**Printing speed**	4	5.09	5.09	1.27	1.9	0.275	2.92
**Layer thickness**	4	5.76	5.76	1.44	2.15	0.238	3.31
**Infill density**	4	130.82	130.82	32.7	48.9	0.001	75.16
**Infill pattern**	4	18.69	18.69	4.67	6.98	0.043	10.74
**Residual error**	4	2.68	2.68	0.67			
**Total**	24	173.05					

**Table 13:** Comparison between predicted and experimental values for different mechanical responses and printing time.

Config	ILSS (MPa) (Predicted/Experimental)	% Error	Max Stress (MPa) (Predicted/Experimental)	% Error	Flexural Modulus (MPa) (Predicted/Experimental)
**1**	2.98/2.01	32.5	20.7/17.5	15.6	694/611
**2**	2.69/1.5	44.2	20.1/19.6	2.5	760/685
**3**	2.87/1.89	34.1	21.9/19.6	10.5	704/668

**Table 14:** Printing parameters of orthoses and associated times.

Orthosis	Extrusion Temp (°C)	Printing Speed (mm/s)	Layer Height (mm)	Infill Density (%)	Infill Pattern	Print Time (h)
**1**	260	50	0.2	99	Grid	34 h
**2**	260	50	0.35	99	Gyroid	21 h 55
**3**	270	60	0.2	100	Cubic	28 h 40
**4**	260	50	0.25	85	Cubic	22 h 21
**5**	270	50	0.3	100	Cross 3D	21 h 28

**Table 15:** Orthosis failure test results.

Orthosis	Breaking Force (N)	Breaking Displacement (mm)
**1**	98.5	147.2
**2**	91.0	136.9
**3**	105.2	169.2
**4**	92.7	159.5
**5**	82.8	100.82

**Table 16:** Digimat simulation results.

Orthosis	Maximum Warpage (mm)	von Mises Stress (MPa)
**1**	9.93	17.33
**2**	6.04	10.72
**3**	5.41	18
**4**	6.98	3.94
**5**	6.41	7.56

**Table 17:** Simulation results for orthosis 3.

Orientation	Maximum Warpage (mm)	von Mises Stress (MPa)
90°	5.41	18
45°	33.61	21.24

**Table 18:** Simulation results for compensated orthosis 3.

Orthosis	Maximum Warpage (mm)	von Mises Stress (MPa)
**Normal model**	5.41	18
**Compensated model**	3.25	12.74

## Data Availability

The raw data cannot be shared at this time as the data are also part of an ongoing study.

## Supplementary Materials

The following supporting information can be downloaded at: https://www.mdpi.com/article/10.3390/polym17141921/s1, Figure S1: DSC thermogram of TREED PLENE 5 PP filament. Figure S2: DSC thermogram of Hifax 3080 PP pellet. Figure S3: Mass loss curve as a function of temperature during TGA. Figure S4: Dimensional variation of PP as a function of temperature. Figure S5: Thermal conductivity of PP as a function of temperature. Figure S6: Young’s modulus of PP as a function of temperature. Figure S7: Specific volume of PP as a function of temperature at P = 0 MPa. Figure S8: Stress-strain curve of PP. Table S1: Summary of thermal properties and crystallinity of PP grades.
