# Supplementary material for: Process Parameters Optimization and Mechanical Properties of Additively Manufactured Ankle–Foot Orthoses Based on Polypropylene
Source: Polymers (Basel). 2025 Jul 11;17(14):1921. doi: 10.3390/polym17141921 (PMC12299998; doi:10.3390/polym17141921)
Supplement: Supplementary file 1 [file polymers-17-01921-s001.zip › polymers-3731625-supplementary.pdf]

Process Parameters Optimization and Mechanical Properties of Additively Manufactured Ankle–Foot Orthoses Based on Polypropylene

Sahar Swesi [1], Mohamed Yousfi [1,*], Nicolas Tardif [2] and Abder Banoune [3]

[1] Université de Lyon, CNRS, UMR 5223, Ingénierie des Matériaux Polymères, Université Claude Bernard Lyon 1, INSA Lyon, Université Jean Monnet, 69621 Villeurbanne Cedex, France; sahar.swesi@insa-lyon.fr
[2] Univ Lyon, INSA-Lyon, CNRS, LaMCoS, UMR5259, 69621 Villeurbanne, France; nicolas.tardif@insa-lyon.fr
[3] Handicap International, Humanité et Inclusion, 69008 Lyon, France; a.banoune@hi.org
* Correspondence: mohamed.yousfi@insa-lyon.fr

Appendix

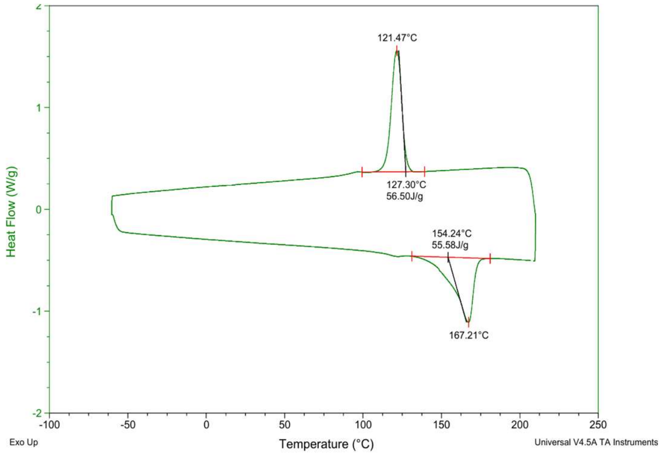

Figure S1 : DSC thermogram of TREED PLENE 5 PP filament.

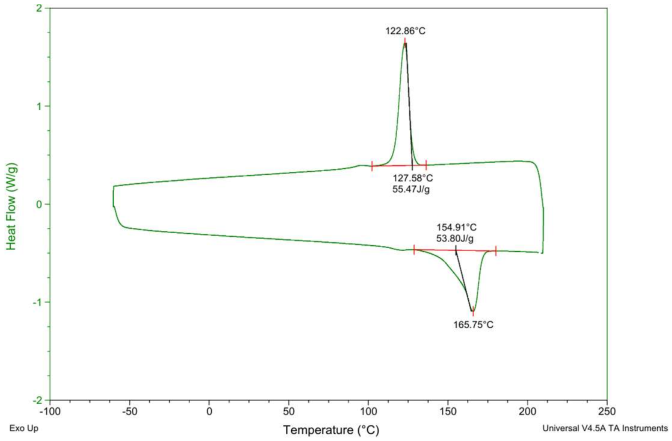

Figure S2 : DSC thermogram of Hifax 3080 PP pellet.

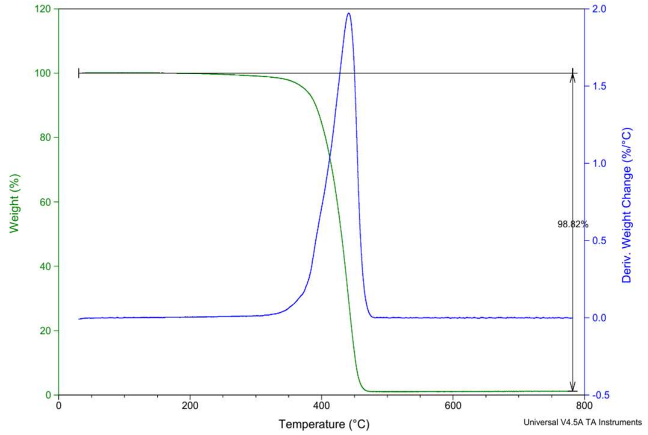

Figure S3 : Mass loss curve as a function of temperature during TGA.

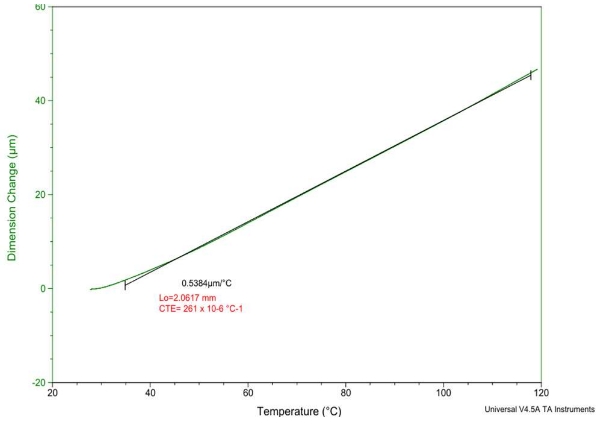

Figure S4 : Dimensional variation of PP as a function of temperature.

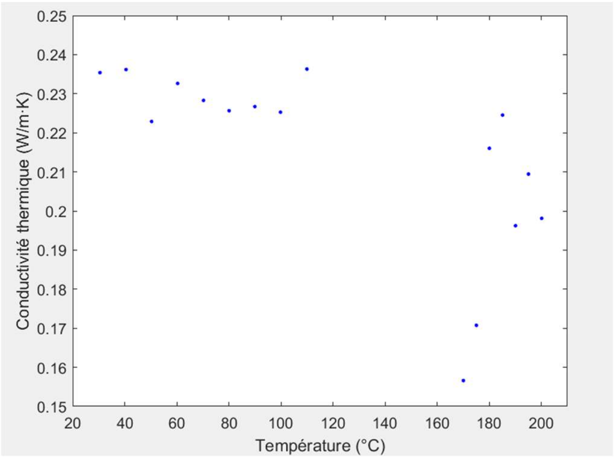

Figure S5 : Thermal conductivity of PP as a function of temperature.

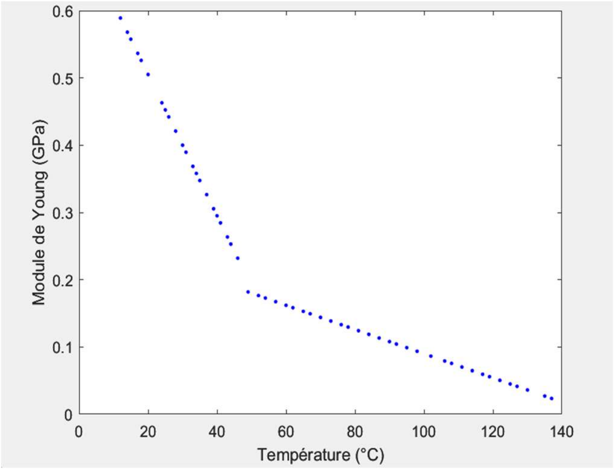

Figure S6 : Young's modulus of PP as a function of temperature.

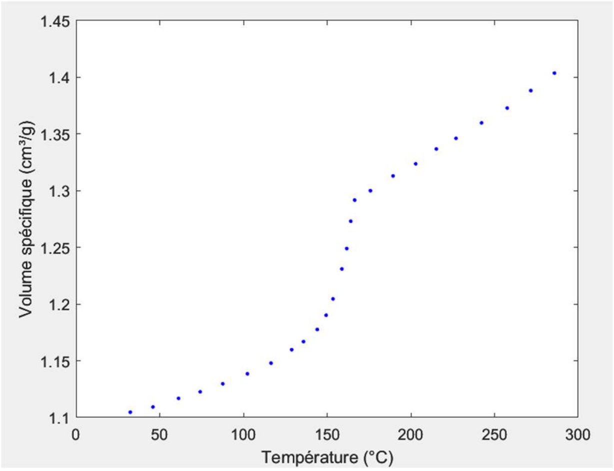

Figure S7 : Specific volume of PP as a function of temperature at P = 0 MPa.

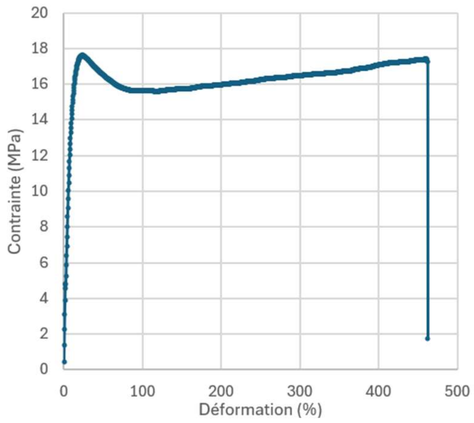

Figure S8 : Stress-strain curve of PP.

Table S1 : Summary of thermal properties and crystallinity of PP grades.

Material	Tm (°C) ± 1 °C	Tc (°C) ± 1 °C	Xc (%) ± 1 %
**TREED PLENE 5**	167	121	27
**Hifax 3080**	166	123	26
